# Vitiligo and tumor response in a patient with amelanotic melanoma undergoing nivolumab treatment

**DOI:** 10.1007/s13691-021-00515-w

**Published:** 2021-10-12

**Authors:** Satoshi Furune, Chiaki Kondo, Yuko Takano, Tomoya Shimokata, Mihoko Sugishita, Ayako Mitsuma, Osamu Maeda, Yuichi Ando

**Affiliations:** Department of Clinical Oncology and Chemotherapy, Nagoya University Hospital, 65 Tsurumai-cho, Showa-ku, Nagoya, 466-8550 Japan

**Keywords:** Amelanotic melanoma, Nivolumab, Vitiligo, irAE

## Abstract

Vitiligo, an acquired depigmenting disorder of the skin that reacts against normal melanocytes, sometimes occurs as an immune-related adverse event in the treatment of melanoma with immune checkpoint inhibitors. It has been known that the occurrence of vitiligo is associated with a favorable therapeutic response in patients with melanoma, but it is not yet clear whether the association also applies to amelanotic melanoma, a minor subtype of melanoma with little or no melanin pigmentation. We report a patient with amelanotic melanoma of the esophagus who responded well to nivolumab treatment. Shortly after the tumor response, vitiligo was found on the patient’s forearms. This case suggests that the occurrence of vitiligo is associated with a favorable response to nivolumab treatment for amelanotic melanoma.

## Introduction

The recent advent of immune checkpoint inhibitors (ICIs) and molecular targeting agents has brought great therapeutic benefit to many patients with melanoma. Vitiligo, an acquired depigmenting disorder of the skin that reacts against normal melanocytes, sometimes occurs in patients with melanoma spontaneously or during the treatment, especially in those who undergo the treatment with ICIs as an immune-related adverse event (irAE) [1]. It has been known that vitiligo is associated with a favorable response to the treatment in patients with melanoma. However, in the case of amelanotic melanoma, a minor subtype of melanoma that has little or no melanin pigmentation, it is not yet clear whether vitiligo plays a predictive role to the response to the treatment. We report a 62-year-old patient with amelanotic melanoma of the esophagus who responded well to nivolumab treatment. Shortly after the tumor response, vitiligo was found on the patient’s forearms.

## Case report

**Fig. 1:**
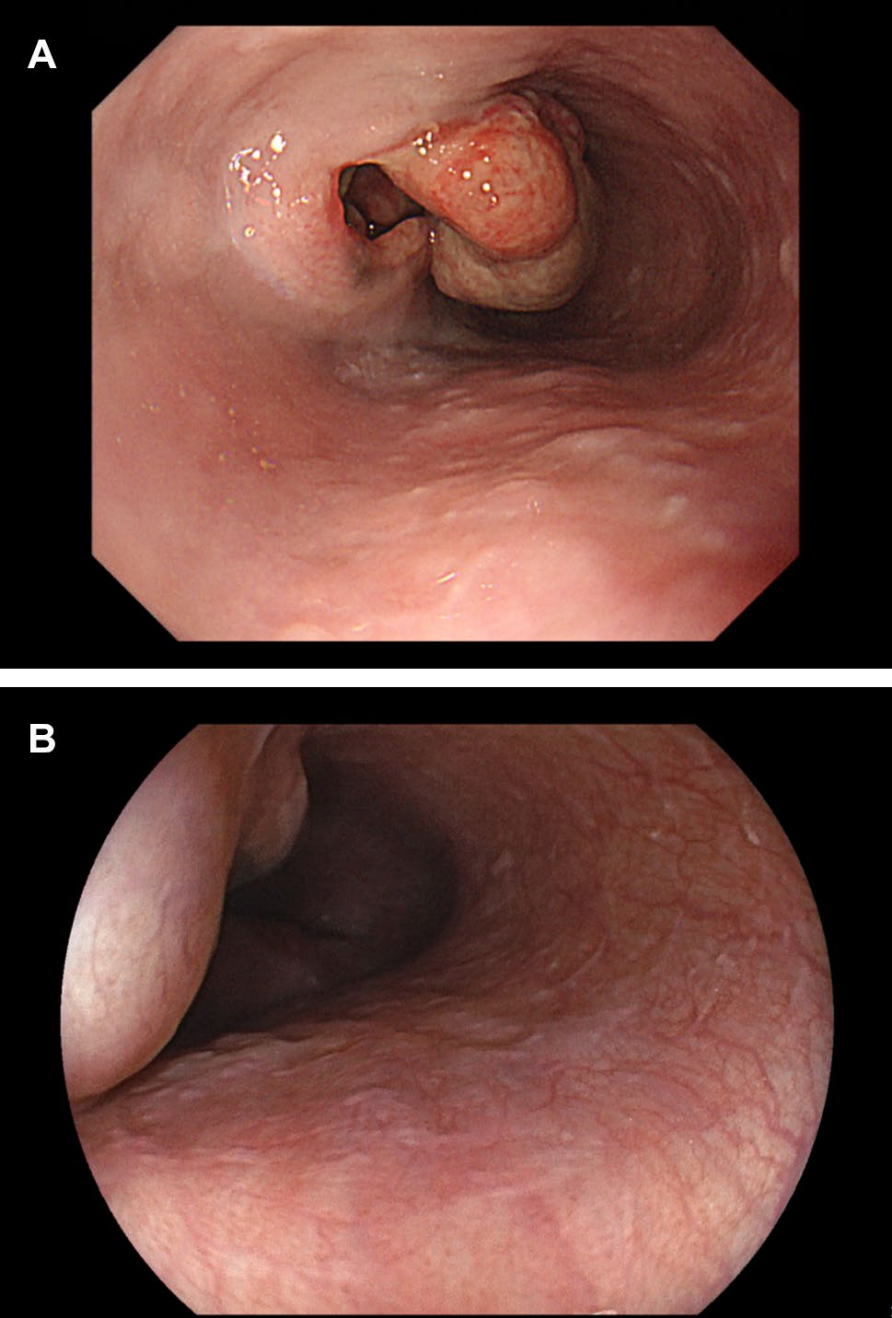
EGD findings before and during treatment. **A** Non pigmented lesion with large ulcer at lower esophagus. **B** Shrinked lesion at lower esophagus after 10th dose

A 62-year-old male patient was referred to our institution for the diagnosis and treatment of an esophageal tumor found by an esophagogastroduodenoscopy (EGD) at a regular health checkup. The patient was asymptomatic, and his alcoholic-related liver disease and diabetes mellitus were both medically well-controlled. The patient underwent endovascular treatment for an abdominal aortic aneurysm five years ago. Physical examination at the initial visit showed neither skin melanoma, skin rash, nor vitiligo on any part of the body. An endoscopic examination demonstrated a 3 cm tumor at the lower esophagus with protruded lesions and ulcers. The proximal edge of the lesion was flat and smooth and looked like a subepithelial lesion. Apparently, the tumor was not pigmented but redness and partly fading. (Fig. 1A). Computed tomography (CT) imaging showed the wall aberrant thickening of the lower esophagus, lymph nodes swelling more than 2 cm at the gastric lesser curvature and minor latent interstitial pneumonitis in the bilateral lungs. A histopathological examination of biopsy specimens from the esophageal tumor reported undifferentiated carcinoma that was positive for Melan A and S-100 protein and negative for cytokeratin AE1/AE3 and p40 on the immunohistochemical (IHC) test. The patient was diagnosed with primary amelanotic melanoma of the esophagus. The tumor cells did not carry *BRAF *^*V600E*^ mutation and the IHC test showed positive (≥ 1%) for programmed death-ligand 1 (PD-L1) expression. Microsatellite instability (MSI) status was not assessed. The subsequent comprehensive genomic profiling test (the OncoGuide^™^ NCC Oncopanel System) identified a somatic mutation of *TP53* gene (C135Y), *PDGFRA* amplification, and a tumor mutation burden (TMB) value of 4.7 mutations/Mb.

**Fig. 2:**
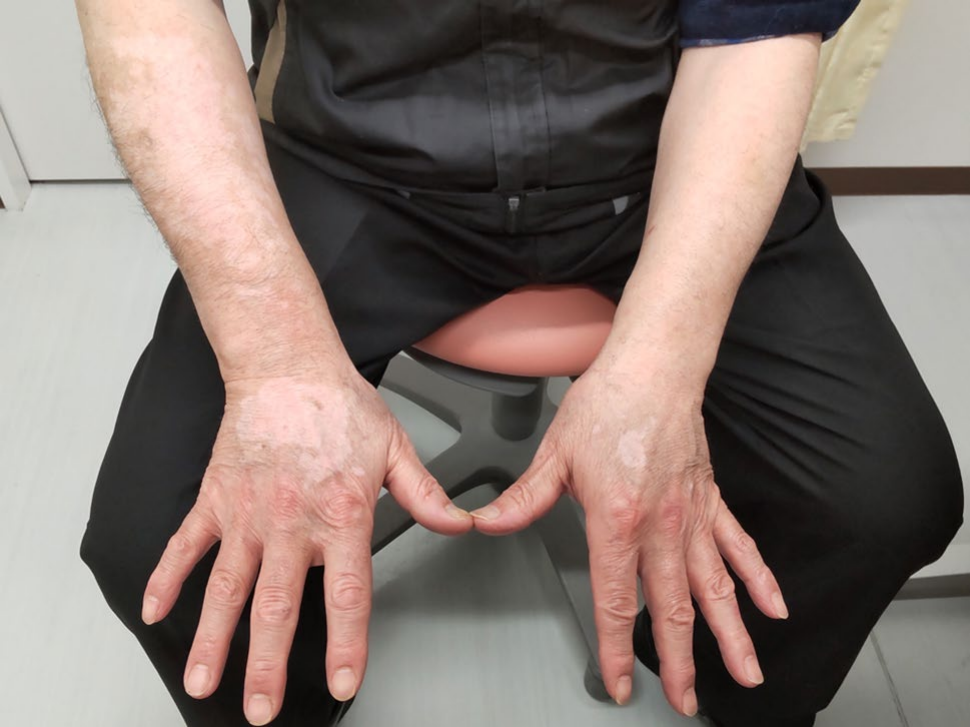
Vitiligo. Vitiligo appeared at both forehand after 11th dose

Initially, we started nivolumab monotherapy at a dose of 240 mg/body every other week and continued at a dose of 480 mg/body once every 4 weeks after the 11th dose. Response evaluation by EGD five months after the start of treatment (after the 10th dose of nivolumab) showed remarkable tumor shrinkage and ulcer healing at the esophageal lesion, indicating a favorable tumor response to the treatment. Skin rash emerged at palms and soles two months after the start of treatment and was easily managed with topical steroid treatment. Two months after the tumor response, 224 days after the start of treatment, vitiligo was found on the patient’s forearms and did not change during and after the treatment (Fig. 2). Both irAE were categorized as Grade 1 according to the Common Terminology Criteria for Adverse Events (CTCAE; version 5.0). Because exacerbation of interstitial pneumonitis was suspected on follow-up chest CT imaging, the patient had to discontinue nivolumab treatment 7 months after the start of treatment. At a follow-up visit 2 months later, there was no evidence of tumor growth on EGD, and vitiligo was apparently unchanged.

## Discussion

This patient with amelanotic melanoma responded well to nivolumab treatment and vitiligo occurred shortly after the response, suggesting that his occurrence of vitiligo would be associated with the response. Amelanotic melanoma, a minor subtype of melanoma that accounts for 10–25% of all the melanoma [2], has little or no melanin pigmentation and does not show typical brown or black color appearance. Generally, amelanotic melanoma exhibits aggressive behavior compared with common cutaneous melanoma and hardly responds to conventional chemotherapy. However, the recent advent of ICIs and molecular targeting agents have brought great therapeutic benefit to many patients with the disease.

Vitiligo sometimes occurs in patients with melanoma spontaneously or during the treatment, especially in those who undergo the treatment with ICIs as an irAE probably because of enhanced immune response and the resulting prolonged survival [3]. It has been known that vitiligo is associated with a favorable response to the treatment in patients with melanoma. In a prospective observational study of patients who received pembrolizumab for melanoma, vitiligo occurred in 17 (25%) of the 67 patients with the time to onset ranged from 52 to 453 (median 126) days. Those patients with vitiligo had significantly better tumor response and survival than those without vitiligo [4]. The predictive role of vitiligo is partly explained by an autoimmune reaction against healthy melanocyte antigen triggered by the treatment, especially ICIs, which destroys melanocytes in the normal skin resulting in the appearance of patchy depigmentation. The vitiligo of this patient occurred on the bilateral forearms, which is consistent with the fact that vitiligo exhibits systematic distribution in most patients with melanoma [5]. In addition, the skin rash is the common dermatologic irAE that occurred together with vitiligo [4].

In the case of amelanotic melanoma that contains little or no melanin pigment, however, it is not yet clear whether vitiligo plays the predictive role to the response to treatment. Although it cannot be completely ruled out that they may have occurred separately, this case provides a piece of evidence that vitiligo is a predictive biomarker of response to treatment in patients with amelanotic melanoma.

PD-L1 expression is a biomarker of the response to treatment with ICI for melanoma. The positivity of PD-L1 expression varies by subtypes of melanoma and the difference between mucosal and cutaneous melanoma is not significant [6]. However, it is unclear to what extent the positive PD-L1 expression in this patient may have contributed to a favorable response. TMB-high, another predictive biomarker, was negative (< 10 mutations/Mb), and MSI status was not evaluated as it was not required to make treatment decisions in this patient.

Melanoma of the esophagus originates from the mucosal tissue and account for only 0.2% of all esophageal tumors [7], and amelanotic melanoma makes up about 10–25% of all melanoma of the esophagus. Therefore, amelanotic melanoma of the esophagus is such a rare disease that its clinical behavior has not been well understood. This case provides an insight into the therapeutic management of this rare disease.

## Abbreviations

ICI: Immune checkpoint inhibitor
EGD: Esophagogastroduodenoscopy
irAE: Immune-related adverse event
CT: Computed tomography
IHC: Immunohistochemical
PD-L1: Programmed death-ligand 1
MSI: Microsatellite instability
TMB: Tumor mutation burden
