# Correction to: Vitiligo and tumor response in a patient with amelanotic melanoma undergoing nivolumab treatment

**DOI:** 10.1007/s13691-021-00517-8

**Published:** 2021-10-26

**Authors:** Satoshi Furune, Chiaki Kondo, Yuko Takano, Tomoya Shimokata, Mihoko Sugishita, Ayako Mitsuma, Osamu Maeda, Yuichi Ando

**Affiliations:** Department of Clinical Oncology and Chemotherapy, Nagoya University Hospital, 65 Tsurumai-cho, Showa-ku, Nagoya, 466-8550 Japan

## Correction to: International Cancer Conference Journal 10.1007/s13691-021-00515-w

In the original publication, the section “Acknowledgments” is not be included. The Acknowledgments section is given in in this correction.

**Acknowledgments** This research was partly supported by AMED under Grant Number JP18ck0106463.

